# Editorial: Intersections of ageing and disability during the COVID-19 pandemic

**DOI:** 10.3389/fsoc.2024.1501580

**Published:** 2024-10-14

**Authors:** Bethany Simmonds, Maria Berghs

**Affiliations:** ^1^Department of Geography and Earth Sciences, Aberystwyth University, Aberystwyth, United Kingdom; ^2^School of Allied Health Sciences, De Montfort University, Leicester, United Kingdom

**Keywords:** ageing, disability, COVID-19, human rights, precarity, care ethics, intersectional feminism

The pandemic exposed the loss of human rights of older and disabled people and illustrated the critical commonalities these two groups have. As sociologists with personal and professional experience of ageing and disability, we were concerned with the proliferation of such “care-less” spaces (Rogers, 2017) and wanted to explore what could be learnt. Considering the general population's positive trajectory in increased life expectancy, not enough has been written about experiences of ageing with, and into, disability. This Research Topic brings together a range of epistemological and methodological perspectives in five papers to understand how to situate a better future of care.

Simmonds' article, “*From bare life and necropolitics to a feminist care ethic: ageism in the COVID-19 pandemic and future directions*,” illuminates how necropolitical decision-making was based on age as a proxy measure for health and was used to determine who received treatment. She explains “exceptional” practices that took place to ration care for older people, particularly in the first wave of the pandemic, using triaging tools in some cases regardless of availability or chances of survival. These practices contravened human rights and challenged equality legislation, yet they are not discrete incidences; rather, they can be seen as part of a continuum which, due to neoliberalization and austerity measures in the United Kingdom, often reduce older people's treatment in the health and social care system to “bare life” conditions outside of legal protection. Simmonds argues that care ethics need to shift from employing universalistic impartial ethical frameworks, like utilitarianism, to guide decisions about care in a detached manner using standardized protocols toward relational, therapeutic, and reciprocal approaches, which integrate the care ethic characteristics of attentiveness, responsibility, competence, and responsiveness within caring networks.

Berghs et al.'s empirical research entitled “*The indignities of shielding during the COVID-19 pandemic for people with sickle cell disorders: an interpretative phenomenological analysis*” discusses the intersecting discriminations of being in a minority ethnic group, ageing, and having a chronic health condition. These intersecting structures of inequality are discussed in relation to the emotional impact they had on this population of people with sickle cell. Specifically, they refer to the fear of being “triaged” and there not being anyone to advocate for them if admitted to hospital and the ableism alongside racism, which has contributed to their condition being placed lower down the “hierarchy of illnesses.” This article also contributes to theorising the concept of time, as how this group experienced time both sped up and slowed down during the lockdowns, and the chronicity of sickle cell did not correlate with a specific temporality, even if some described pandemic time being dissimilar to their everyday lives. Interestingly, findings also point to an inverse relationship between age and disability; when people aged, they moved from acute to less disabling chronic illness. Finally, a major theoretical contribution of this article was related to the conceptualisation of the “ethics of crisis.”

The theme of temporality was also present in McFarland et al.'s work, “*Greying arts access: crafting creative online programming to promote older adults' artistic engagement in and beyond pandemic time*.” This participatory designed research based in Canada contributes perspectives on some of the unintended consequences of the pandemic, which enhanced access to the arts for people growing older with, or into, disability via online technologies whilst also highlighting the need for technological advancement to be designed *with* not just *for* older and disabled groups. Their innovative findings point to a shift from “pandemic time,” when virtual spaces, adapted for wider society, improved the inclusivity of older and disabled people. Their findings also point to ways in which digitalisation can both include older people in cultural artistic engagement whilst simultaneously excluding those with less digital literacy. This segues nicely into König and Seifert's article, “*Internet usage, frequency and intensity in old age during the COVID-19 pandemic—a case study for Switzerland*.” König and Seifert analyzed large-scale survey data reporting on internet usage in later life and found that, although a growing proportion of older adults use the internet, the picture is complex. Gender differences continue to exist (women use it less) but the gap is decreasing, and class-based indicators (such as education and employment) have shaped usage. Further, interestingly they did not find that COVID-19 had any significant influence on digital adoption for older age groups despite its perceived importance in ensuring social bonds during pandemic-related restrictions on movement. Therefore, their findings point to a focus on including older people and making technology accessible.

A theme of resistance is exemplified by Alnamnakani's article entitled “*A narrative case study of an older disabled Muslim woman during the COVID-19 pandemic in the UK*.” This gives an in-depth account of a disabled older Muslim woman's experience of discrimination during the pandemic. This powerful piece skilfully illustrates the indirect impact that COVID restrictions had on experiences of disablism, racism, and sexism related to public transport. This article makes an important theoretical contribution which is the assertation of agency over structure. Although Zora refused to be labeled as a victim, instead calling herself “brave” for acting against her abuser and addressing the collective safety of women, the incident still had a lasting effect on her willingness to use public transport alone. This article illustrates how political decisions in shaping social spaces that place women in vulnerable positions (particularly women who are older, minority ethnic, and/or disabled) need consideration.

This Research Topic illustrates how easily “care-ethics” can be suspended, allowing for “care-less” spaces (and times) to proliferate (Rogers, 2017). Demonstrating that vulnerability is fluid and existential, for instance, the pandemic revealed how political decision-making produced precarious groups who were then failed in terms of care (Simmonds, 2021). These articles offer in-depth original epistemological and methodological insights, evidencing the need for an “anti-ableist and anti-ageist ethics of care” to ensure the maintenance of human rights and dignity in society.
